# The “practice track” – How can teaching content related to outpatient healthcare be integrated into medical studies? Learning objectives, conception and implementation of a specialized voluntary program

**DOI:** 10.3205/zma001323

**Published:** 2020-04-15

**Authors:** Nadja Becker, Linda Barthen, Lia Pauscher, Ferdinand M. Gerlach, Robert Sader, Gisela Ravens-Taeuber

**Affiliations:** 1 Goethe University Frankfurt am Main, Institute of General Practice, Frankfurt/Main, Germany; 2 Goethe University Frankfurt am Main, Dean's Office of the Faculty of Medicine, Frankfurt/Main, Germany

**Keywords:** outpatient health care, family medicine, shortage of doctors, medical studies, curriculum

## Abstract

The “practice track” (PAT) at Goethe University Frankfurt provides students with the opportunity to focus on outpatient care during their medical studies. The aim of this article is to describe the objectives, conception and implementation of the program.

At the Institute of General Practice, a new teaching concept has been developed in cooperation with the Dean’s Office of the Faculty of Medicine at Goethe University and further partners. Medical students generally receive their training in highly specialized hospitals. However, the new concept will allow them to gain a practical insight into the outpatient care provided by physicians in private practice during their medical studies. Every year, 15 interested students will be able to participate in the longitudinal program, which includes internships, seminars and mentoring sessions. In the three current PAT cohorts, all 45 places have been taken up, and the first cohort has now completed the program. In addition to practical skills in the disciplines of family medicine, internal medicine, surgery, gynecology and pediatrics, it has been possible to show students the full scope of ambulatory health care. However, legal limitations to the implementation of the program in registered medical practices have meant that some parts of it could only be carried out voluntarily.

Against the background of the current and future situation in health care, it makes sense that registered physicians in private practice should teach medical students about outpatient care during their medical studies. In order to establish such programs and permit their complete integration into the medical curriculum, it is essential that the necessary changes are made to medical licensing regulations.

## Zusammenfassung

Der „Praxis-Track“ (PAT) an der Goethe-Universität Frankfurt am Main bietet Studierenden die Möglichkeit, schon während ihres Medizinstudiums einen Schwerpunkt auf die ambulante Patientenversorgung zu legen. In diesem Artikel sollen erstmalig Ziele und Konzeption sowie die Umsetzung des Programms beschrieben werden.

Im Institut für Allgemeinmedizin wurde in Zusammenarbeit mit dem Studiendekanat des Fachbereichs Medizin der Goethe-Universität und weiteren Fachvertretern ein neues Lehrkonzept entwickelt. Dieses ermöglicht Medizinstudierenden schon während ihrer Ausbildung – die überwiegend in hochspezialisierten Kliniken stattfindet – vermehrt praktische Einblicke in die ambulante Tätigkeit niedergelassener Ärzte. Jährlich können 15 interessierte Studierende an dem longitudinalen Programm teilnehmen, das Praktika, Seminare und ein Mentoringprogramm umfasst. In den aktuell drei Kohorten des PAT wurden alle 45 Plätze belegt, die erste Kohorte hat das Programm erstmals vollständig durchlaufen. Es ist gelungen, den Studierenden neben praktischen Fähigkeiten in den Fächern Allgemeinmedizin, Innere Medizin, Chirurgie, Gynäkologie und Pädiatrie in besonderem Maße die Bandbreite sowie das System der ambulanten Versorgung zu vermitteln. Die Durchführung in vertragsärztlichen Praxen stieß an studienrechtliche Grenzen, daher sind einzelne Programmbestandteile nur auf freiwilliger Basis möglich.

Ausbildungsinhalte zur ambulanten, vertragsärztlichen Versorgung in das Medizinstudium zu integrieren, erscheint vor dem Hintergrund der aktuellen und künftigen Versorgungsrealität sinnvoll und notwendig. Eine Voraussetzung für die Etablierung solcher Programme ist die Schaffung der entsprechenden studienrechtlichen Rahmenbedingungen, um eine vollumfängliche curriculare Integration des Programms zu ermöglichen.

## 1. Introduction

During medical studies, practical training occurs primarily in the inpatient sector and much of it in highly specialized university hospitals. In contrast, the majority of medical care provided to the general population in Germany takes place in the outpatient sector [1]. Currently, around 50 percent of physicians work in inpatient facilities, about 40 percent in the outpatient sector, and the remaining 10 percent in other areas [2], [3]. In reality, 19.5 million cases are treated in the inpatient sector, compared to more than a billion patient contacts in outpatient medical practices [4], [5]. Furthermore, the proportion of treatment occurring in the outpatient sector is on the increase, partly because of medical-technological progress, but also because services can be provided more efficiently in this setting [6]. In addition to structural change, the healthcare system must also deal with the challenges presented by demographic developments: a consequence of a rise in life expectancy is an increase in chronic diseases and multimorbidity. This, in turn, leads to an increase in complexity, and a greater need for collaboration within the healthcare system [1]. At the same time, the medical fraternity is also changing: an increase in the average age of physicians, part-time work, and shortcomings in the allocation of medical resources, are intensifying the threat of a shortage of physicians, particularly in family medicine and in rural areas [1], [2], [7].

New teaching concepts are required to deal with the discrepancy between medical training and the actual situation facing the health care system by strengthening the focus on outpatient medical care and the importance of family medicine. This is also the aim of the “medical studies masterplan 2020”, which has recently been passed by Federal and State governments. Important elements of the plan include increasing the importance attached to educating students in outpatient care and everyday disease patterns, and strengthening the role played by family medicine. In an increasingly complex healthcare system, the objective is to help students gain a basic understanding of patient-oriented cooperation between family and specialist physicians, as well as with other health professionals. Flanked by additional measures, this teaching content should help motivate students to set up their own private practices [8]. The results of a student survey commissioned by the *Kassenärztliche Bundesvereinigung* show that the willingness of students to work as registered physicians in the outpatient sector is high – almost 70 percent of respondents said they could imagine working in the outpatient field. However, 53 percent of respondents said they considered themselves poorly informed in matters relating to the conditions and requirements of work in outpatient healthcare. A possible explanation for this is the low level of contact with physicians working in the outpatient sector, who are therefore unable to serve as sources of information and role models. Although 37 percent of respondents said they could imagine specializing in family medicine, only around 10 percent of all nationwide specialist certifications occurred in the discipline in the years 2017 and 2018 [2], [3], [9]. 

The number of structured, longitudinal family medicine support programs in Germany has steadily increased in recent years [10]. However, they differ considerably in terms of structure and content and are still relatively new compared with similar programs abroad [10], [11]. Teaching projects at home and abroad that are aimed at supporting young (rural) doctors in family medicine have had a positive effect on medical students’ interest in family medicine and in their pursuing a career in the field, even when taking into account that preselected target groups may have led to some bias. Important contributing factors to an increase in their interest are early and continuous thematic involvement in the field, and the existence of professional role models [7], [12], [13], [14].

Against this background, the “practice track” (PAT) program was developed at Goethe University Frankfurt am Main. Early on in their studies, medical students that are interested in doing so can get to know outpatient healthcare and the teams responsible for delivering it during the clinical semesters of their studies, and they can specialize in the field. In order to integrate and complement existing course structures and ensure that the additional work involved for administrative personnel and participating students is kept to a minimum, the concept also includes elements of the medical curriculum. The present article will describe the objectives, conception and implementation of the PAT for the first time. 

## 2. Project description

### 2.1. Teaching objectives

Participating students will have the opportunity to become thoroughly familiar with outpatient healthcare during their studies. On the one hand, the focus will be on patients and practices, and on teaching students about the most frequent diseases and reasons consultations are sought in the involved medical disciplines. On the other hand, they will receive an intensive introduction into what pursuing a career in outpatient care entails, and they will get to know the special features and challenges of outpatient and cross-discipline healthcare (cooperation with other medical specialists and healthcare professions, the administrative framework, career perspectives). Depending on their own individual interests, the students will get an idea of what it means to work in the outpatient sector. Importance is also attached to providing students with individual support, and to helping and encouraging them to plan their own careers. 

#### 2.2. Conception 

The conceptual development was partially based on a survey of medical students at Goethe University in which, although demonstrating considerable interest in family medicine, students also said they did not consider themselves well prepared to work in the field. By means of a search both in the literature and online, information was gathered on inter-/national training programs used to support family medicine during medical studies [11]. In the conceptual phase, which lasted around one year, a training plan was developed in cooperation with the Dean, the Dean’s Office, and our partners in the relevant fields. The plan moved outpatient care into the focus of medical studies, while at the same time keeping administrative work to a minimum. Topics considered to be of importance during the implementation phase included suitable information and promotion measures, the selection of participants, the content of the accompanying seminars and mentoring sessions, and the selection of suitable academic teaching practices.

#### 2.3. Concept

The program is longitudinal and participation is voluntary at any time during the clinical phase of medical studies (six semesters or three years) from the first clinical semester onwards. Fifteen students can participate each year, bringing the total number to 45 during the clinical study period. 

**Figure 1:**
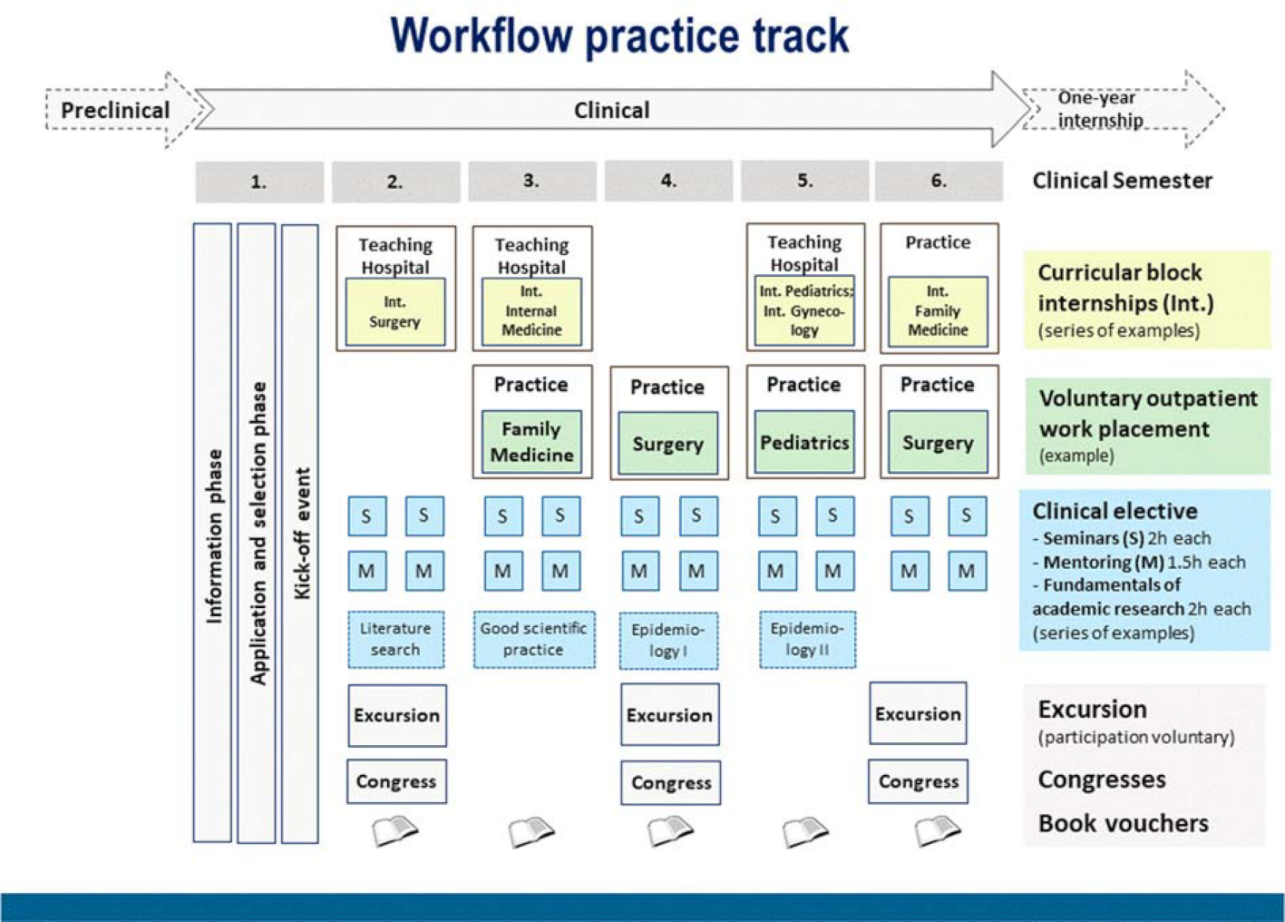
Overview of workflow and components of practice track

The program is made up of three main elements (see figure 1 (Fig. 1)):

1. Curricular block internships at selected hospitals
2. Voluntary outpatient work placements at registered private practices
3. Seminars and mentoring sessions (clinical elective)

##### 2.3.1. Curricular block internships

Participants complete curricular block internships in internal medicine, surgery, gynecology and pediatrics at selected, small teaching hospitals that provide basic and standard care. The principal aim is that they should gain experience of working in (emergency) outpatient units. In the same way as other medical students, the participants will complete the curricular block internship in family medicine in selected family practices. During these five internships, the participants will be confronted with a wide range of diseases and patients, and they will develop their own practical skills. The allocation of participants will take place in cooperation with the Dean’s Office of the Faculty of Medicine. 

The original intention was that PAT participants should complete their block internships in the practices of medical specialists in the relevant fields, rather than in teaching hospitals. This would have permitted the registered physician sector to become an integral part of curricular, practical training. However, the plan failed for legal reasons. On the one hand, medical licensing regulations (ÄApprO) stipulating that a fixed number of training hours must be completed (“bedside instruction”, defined as teaching at occupied beds beyond midnight) could not have been formally fulfilled. On the other hand, the faculty might also have violated legal capacity restrictions (leading to an increase in the number of study places) [https://www.gesetze-im-internet.de/_appro_2002/BJNR240500002.html, [http://www.vhw-bund.de/DOCS/RECHT/KapVHE1994.pdf]. Consequently, the students must continue to complete their block internships in teaching hospitals. However, in order that they can gain thorough experience of the range of medical specialties that provide outpatient care, they are encouraged to complete voluntary outpatient work placements in specialist practices. 

##### 2.3.2. Voluntary outpatient work placements

Outpatient work placements are voluntary and can be completed at selected teaching practices with trained specialists in family medicine, surgery, gynecology and pediatrics. The practices are chosen according to formal criteria to ensure that subject-specific learning objectives are met (subject-specific services such as regular operations taking place on site in teaching practices for surgery or as an attending physician at a hospital; joint practices wherever possible, consulting hours five days a week, good connections to public transport). The practices are written to and, if they respond, individually advised as to what the program would entail. Participating teaching physicians are introduced to the organization of the program and take part in a workshop on teaching. The work placements last a full week and take place on a 1-to-1 basis (the teaching doctors receive an expense allowance of €125 per week). Special learning objective catalogues developed in cooperation with lecturers in the relevant subjects help the teaching physicians and students define the content of the placements. The aim of the work placements is that the students should experience the entire range of work that the medical specialty involves, get to know the structure and organization of the practice, and gain practical experience. The participants may complete four one-week work placements, of which a maximum of two can be in one subject area. In consideration of the extra work involved, the students receive an expense allowance of €150 per week.

##### 2.3.3. Seminars and mentoring sessions

The accompanying seminars and mentoring sessions cover the requirements of the compulsory elective in the clinical phase of medical studies. The series of seminars (two seminars per semester from the 2nd-6th clinical semesters) provide information on the medical outpatient sector in Germany – topics are, for example, an overview of the German healthcare system, career prospects in private practice, health care models in palliative medicine, dealing with multimorbidity and polypharmacy, the work of medical professions and practice teams in the future, and innovative care models. Furthermore, students can suggest topics that they would like to cover in seminars. Students also complete four selected modules from the Doctoral Program of the Medical Faculty of Goethe University, in which they are taught the fundamentals of working in academic research. 

Mentoring is carried out in small groups of seven or eight students, to which two mentors are allocated. The mentors are young physicians undergoing specialist training. The choice to involve young physicians was made because they recently completed their medical studies and are still aware of what is important, while at the same time being familiar with the current situation in specialist training. The mentoring sessions will take place twice per semester. The topics can be selected by the participants themselves and generally cover individual study organization and career planning (clinical traineeship, one-year internship, doctoral studies, starting work, training to be a medical specialist, additional qualifications, work-life balance and many others). 

Further to the elements of the program described above, participants will also receive a book voucher worth €50 per semester, as well as the opportunity to be reimbursed for the costs of attending congresses that are of relevance to the program (max. €200 per year). Moreover, they also have the option of participating in an annual excursion during which they can become acquainted with innovative healthcare models on site. These include new types of cooperation in outpatient care and involve various medical professions.

##### 2.3.4. Announcement of the program and selection of participants 

At the beginning of the first clinical semester, all students will be invited to an information event at which they will be told about the possibility to participate in the voluntary program. The program will also be promoted, and responsible employees at the Institute of General Practice will be available to answer any questions. 

Applications will be submitted in written form on the basis of a prepared questionnaire. In addition to providing personal details, applicants will be asked to explain why they are interested in participating in the program and what they expect of it. To assess the application documents, points will be allocated according to selection criteria that have been described in the literature as correlating positively with the choice to pursue a career in family medicine. These include, for example, biographical factors (rural background, completed professional training), job expectations or perceptions of a physician’s work, and personal experience of family medicine [15], [16]. The allocation of places will be transparent and depend on the number of points. In case of a tie, a random draw will determine participation.

At the end of the first clinical semester, the program will begin with a kick-off event. This will enable participants and instructors to get to know each other and the organization of the program. 

#### 2.4. Evaluation

The concept used to evaluate PAT will involve the evaluation of both processes and results [17]. In order to ensure process management is optimized, and that further development and corrective measures are taken where necessary, the implementation and execution of the program will be continuously evaluated. All elements of the program will be assessed. Furthermore, a long-term evaluation of results will take place, which will include a graduate destination survey. In this way, the influence of PAT on career aspirations and later work as a doctor will be explored. 

## 3. Preliminary results

It has been possible to develop a varied program that helps participants learn practical skills and acquire theoretical knowledge that complements the existing medical curriculum. Voluntary outpatient internships permit participants to gain structured insights into outpatient care. At the same time, the short duration and the selection of teaching practices mean that organizational work for participants is kept to a minimum. The newly developed seminars and mentoring sessions and the excursion provide a framework that permits participants to obtain a thorough introduction to careers in the outpatient sector. 

The PAT was offered for the first time in the winter semester of 2016/2017. All of the 15 available places were taken up (there were 15 applicants). The first cohort completed the program in the summer semester of 2019. During the winter semesters of 2017/2018 and 2018/19, the 15 available places were also taken up, whereby the number of applicants was 32 and 19 respectively. The participants form a heterogeneous group in terms of biographical data (see table 1 (Tab. 1)).

The implementation process showed that particularly the integration of registered teaching practices and the organization of seminars and mentoring sessions (schedule coordination, recruitment of suitable lecturers) were extremely time consuming. The need to support the students and provide individual arrangements (taking off semesters to study abroad/doctoral studies, catching up in case of absence etc.) made it necessary for someone to be available to help. Sufficient personnel resources are therefore necessary to ensure that the program is well organized. The estimated need for a research assistant at 80% of a full-time position is appropriate to manage three cohorts of 15 medical students. Grants from the Faculty of Medicine and the University were initially secured to finance the program provisionally. In the meantime, the *Kassenärztliche Vereinigung* Hessen has agreed to become a long-term partner in the project. 

## 4. Discussion

The PAT provides the opportunity to focus on outpatient care during medical studies, thus fulfilling one of the major demands of the “masterplan medical studies 2020”. The aim of the masterplan is that medical studies in Germany should be restructured to take into account both the need to intensify academic training, and the complexity of the current healthcare situation [8]. 

In 2002, amendments to the ÄApprO licensing act led to reforms in medical studies and to more practical training content [https://www.gesetze-im-internet.de/_appro_2002/BJNR240500002.html]. The reforms included strengthening the coverage of family medicine at university (through the creation of professorships in general practice at numerous medical faculties, for example, as well as the introduction of a compulsory internship in the discipline), improved integration of theoretical/practical knowledge, as well as numerous teaching projects and initiatives aimed at improving the teaching of practical skills (e.g. skills lab) [18]. Nevertheless, medical students interested in focusing on a specific field can only do so to a limited degree. Furthermore, when planning the new teaching project, many limitations had to be faced that result from current medical licensing regulations [https://www.gesetze-im-internet.de/_appro_2002/BJNR240500002.html], http://www.vhw-bund.de/DOCS/RECHT/KapVHE1994.pdf]. As a result, it is currently impossible to strengthen the integration between registered outpatient practices and compulsory teaching courses. The compulsory number of hours receiving “bedside instruction” prescribed in the ÄApprO cannot be formally implemented in the outpatient sector, even if the same content could be taught with the aid of outpatients, and students could benefit from the additional advantage of an introduction to a large number of relevant diseases. In our opinion, these aspects should urgently be taken into consideration and the implementation of the masterplan linked to a reform of the licensing regulations. In this way, medical faculties would have greater opportunities to involve registered outpatient practices in the medical curriculum. Initial experiences gained with the project have shown that both students and registered physicians in private practice are very interested and committed to the idea that students should be familiarized with the considerable diversity of outpatient healthcare. To achieve this it will be necessary, on the one hand, to create a legislative framework that permits cooperation between medical faculties and outpatient healthcare facilities/registered medical practices without capacity restrictions. On the other hand, it is necessary to embed teaching content and courses in the field of outpatient healthcare in the ÄApprO, and perhaps make them obligatory. If an outpatient component of the curriculum were obligatory, it would be possible for programs such as PAT to help in its implementation by sharing its valuable experience – for example with regard to cooperation with specialist medical practices, the provision of didactical support for teaching physicians, the preparation of specific learning content in the outpatient field, and the thematic conception of a series of seminars on relevant aspects of outpatient healthcare. 

The career plans and expectations of young doctors will play an important role in the preparation of new supporting initiatives. A 2012 survey of students showed that regardless of gender, finding an appropriate balance between work and leisure time and profession and family is very important to young physicians, with career and earnings playing a subordinate role. Furthermore, everyday medical work should involve a high degree of autonomy and diversity, as well as good interdisciplinary cooperation and a good working atmosphere [19]. The design of PAT takes such topics into consideration by choosing the content of the clinical elective accordingly – in the accompanying seminar, physicians in private practice describe their everyday work and report on the diseases they are confronted with, as well as integration with other medical professions. Topics such as career planning and expectations can be dealt with intensively during mentoring sessions. Mentoring programs are considered to be one of the most important instruments used to support students seeking to pursue a career in medicine and are being used increasingly at medical faculties in Germany. The results of an evaluation demonstrate that participating medical students show high acceptance and levels of satisfaction with mentoring programs [20], [21]. Furthermore, the optional annual excursion provides students with the opportunity to become familiar with a wide variety of existing care concepts and working models.

Medical students appreciate the practical orientation of their studies, but still see potential for improvement in the fields of knowledge transfer, structured and systematic working and the promotion of social skills [22]. With its intensive practice and patient orientation and a focus on outpatient care, programs such as PAT can make an important contribution towards teaching young medical students how to integrate their theoretical and practical knowledge and develop the necessary skills to deal with patients in everyday medical practice. 

## 5. Conclusion

In view of the current healthcare situation and expected care needs in the future, it is absolutely essential that medical students are encouraged to work as registered physicians in outpatient care and that they are prepared accordingly. The structured and longitudinal conception of PAT meets these demands. In order to raise the attractiveness of such programs, it is also important that teaching content relating to outpatient care be comprehensively integrated into the medical curriculum. Over the long term, it is desirable that teaching content relating to outpatient care becomes an integral part of medical studies for all medical students.

## Acknowledgements

We would like to thank the Faculty of Medicine and the Förderfonds Lehre at Goethe University Frankfurt, and the Kassenärztliche Vereinigung Hessen for their funding and support in realizing the “practice track” teaching project. 

We would also like to thank the Dean’s Office of the Faculty of Medicine at Goethe University Frankfurt for its support in the allocation of participating students. 

We are grateful to Phillip Elliott for translating the manuscript into English.

## Competing interests

The authors declare that they have no competing interests. 

## Figures and Tables

**Table 1:**
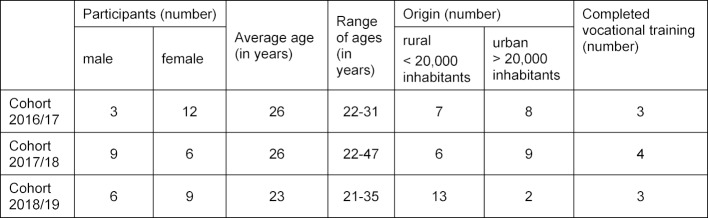
Biographical data of student participants in the practice track cohorts
